# A simple and flexible high-throughput method for the study of cardiomyocyte proliferation

**DOI:** 10.1038/s41598-019-52467-0

**Published:** 2019-11-04

**Authors:** Abigail C. Neininger, J. Hunter Long, Sophie M. Baillargeon, Dylan T. Burnette

**Affiliations:** Department of Cell and Developmental Biology, Vanderbilt University, Nashville, TN 37232 USA

**Keywords:** Cell biology, Cell division

## Abstract

Cardiac muscle cells lack regenerative capacity in postnatal mammals. A concerted effort has been made in the field to determine regulators of cardiomyocyte proliferation and identify therapeutic strategies to induce division, with the ultimate goal of regenerating heart tissue after a myocardial infarct. We sought to optimize a high throughput screening protocol to facilitate this effort. We developed a straight-forward high throughput screen with simple readouts to identify small molecules that modulate cardiomyocyte proliferation. We identify human induced pluripotent stem cell-derived cardiomyocytes (hiCMs) as a model system for such a screen, as a very small subset of hiCMs have the potential to proliferate. The ability of hiCMs to proliferate is density-dependent, and cell density has no effect on the outcome of proliferation: cytokinesis or binucleation. Screening a compound library revealed many regulators of proliferation and cell death. We provide a comprehensive and flexible screening procedure and cellular phenotype information for each compound. We then provide an example of steps to follow after this screen is performed, using three of the identified small molecules at various concentrations, further implicating their target kinases in cardiomyocyte proliferation. This screening platform is flexible and cost-effective, opening the field of cardiovascular cell biology to laboratories without substantial funding or specialized training, thus diversifying this scientific community.

## Introduction

**Figure 1:**
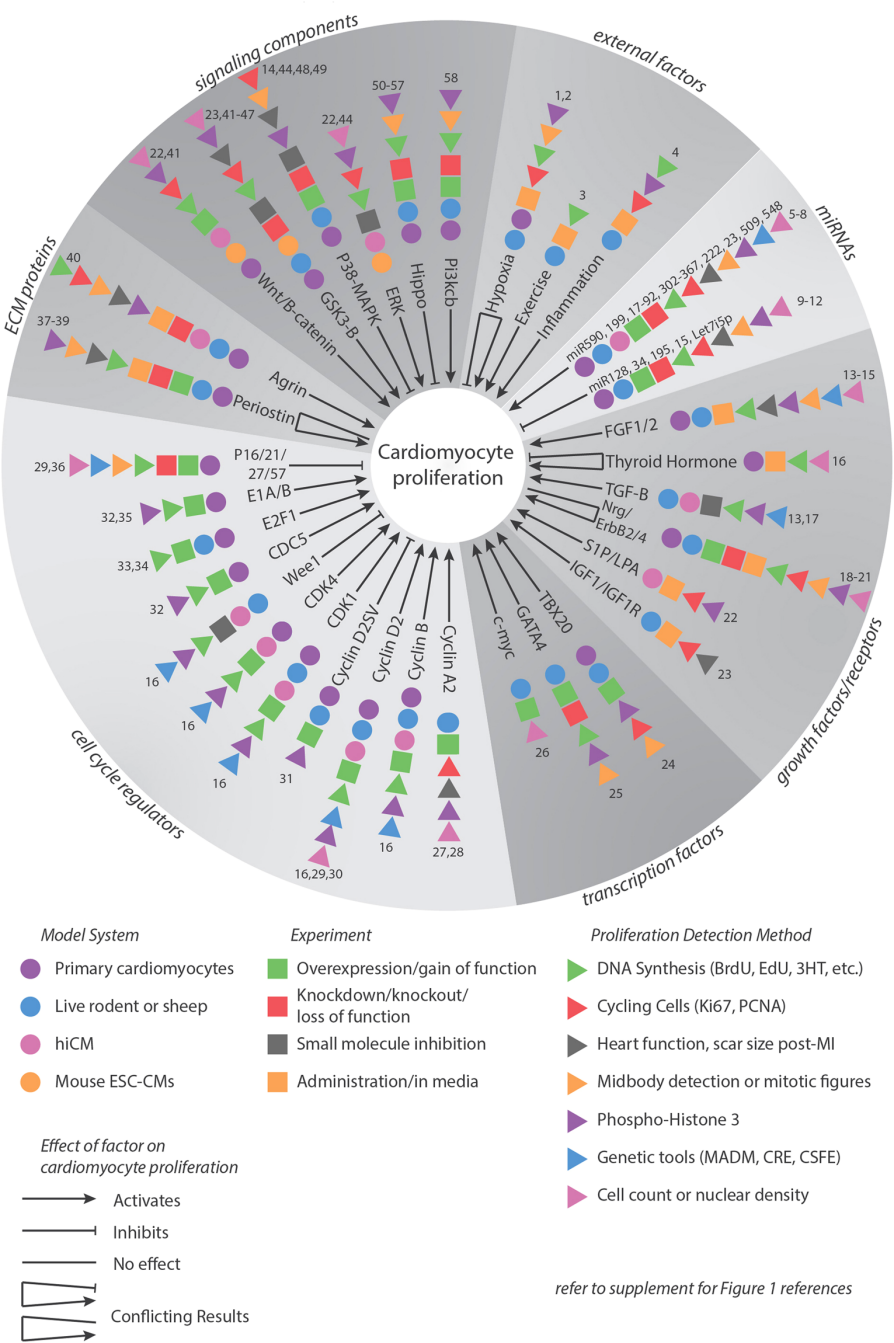
Factors involved in cardiomyocyte proliferation. A brief review of factors identified in rodents or using human iPSCs as regulators of cardiomyocyte proliferation. Circles, squares, and triangles show the model system used, experiment, and proliferation detection method, respectively. Arrows show activating factors, blunt arrowheads show inhibitory factors, and lines show factors determined to have no effect. A combination of two types of arrows for the same factor shows conflicting results. Numbers denote the studies for each factor, which can be found in the supplementary references.

Cardiomyocytes are the essential muscle cells which drive the beating of the heart. These cells display a balance between hyperplastic growth by cell division and hypertrophic growth by cell enlargement during development. However, after birth, mammalian cardiomyocytes exit the cell cycle and rely solely on hypertrophic growth^1^. As a result, cardiomyocytes lost due to injury cannot be replaced. There has been a concerted effort to find factors that induce cardiomyocytes to reenter the cell cycle and successfully undergo cell division. Identification of these factors would facilitate the ultimate goal of regenerating heart muscle *in vivo*, so the heart can heal from injury. Overexpression of positive cell cycle regulators or transcription factors or deletion of negative cell cycle regulators in rodent models has been shown to induce proliferation (summarized in Fig. 1). However, there is a limit to the amount of protein that can be overexpressed in a cell due to proteasome-mediated degradation of the excess protein^2^. Excitingly, a recent study has circumvented this by demonstrating a combinatorial approach utilizing both genetic manipulation and small molecule inhibitors which induced cell division in post-mitotic murine cardiomyocytes *in vivo*^2^. This treatment also improved cardiac function after a myocardial infarction, proving to be a promising approach to regenerate cardiomyocytes *in vivo*. Thus, identifying additional small molecules that induce cardiomyocyte proliferation could be beneficial for future combinatorial therapies.

Current technologies lend themselves to high-throughput screening to identify bioactive small molecules. Such a screening platform needs to be robust, sensitive, and large-scale, with a clear readout. There are several *in vitro* model systems that could be considered for such a screen, each with unique advantages and disadvantages. Cardiomyocytes can be isolated from rodents and represent mature, adult cardiomyocytes. However, these primary rodent cardiomyocytes can only beat in culture for a few days, making them unsuitable for long-term screens. Beating can be inhibited, making these cells will last longer; however, they will dedifferentiate to a more immature state^3^. In addition, the isolation protocols can be variable, resulting in cell populations that differ between batches and laboratories. As such, we believe human induced pluripotent stem cells differentiated into cardiomyocytes likely to be a robust model system. These cells can be maintained in culture for weeks to months and beat continuously, facilitating long-term screens. Although these cardiomyocytes are more transcriptionally similar to embryonic cardiomyocytes, this immature state maintains a slight proliferative potential that can be modulated by small molecules^4^. The long-term goal of many such studies are to test compounds in whole animals, such as with a high-throughput zebrafish screen or a more low-throughput treatment in rodents given a myocardial infarction.

A second major consideration in developing a screening platform is accessibility to researchers from a wide range of disciplines. This requires a straight-forward procedure with a simple and quantitative output. The cost of such a screen also needs to be minimized as the initial investment into a project is often a hurdle for laboratories entering a new field of study. Taking these considerations, here we report a strategy for identifying small molecule regulators of cardiomyocyte proliferation. We use human iPSC-derived cardiomyocytes (hiCM) and relatively low-magnification microscopy to monitor the number of nuclei over time. This has allowed us to screen a library of small molecules to identify compounds that both increase and decrease proliferation. This procedure is straight-forward, flexible, and cost-effective.

## Results

### Identifying hiCMs as a model system for cardiomyocyte proliferation

**Figure 2:**
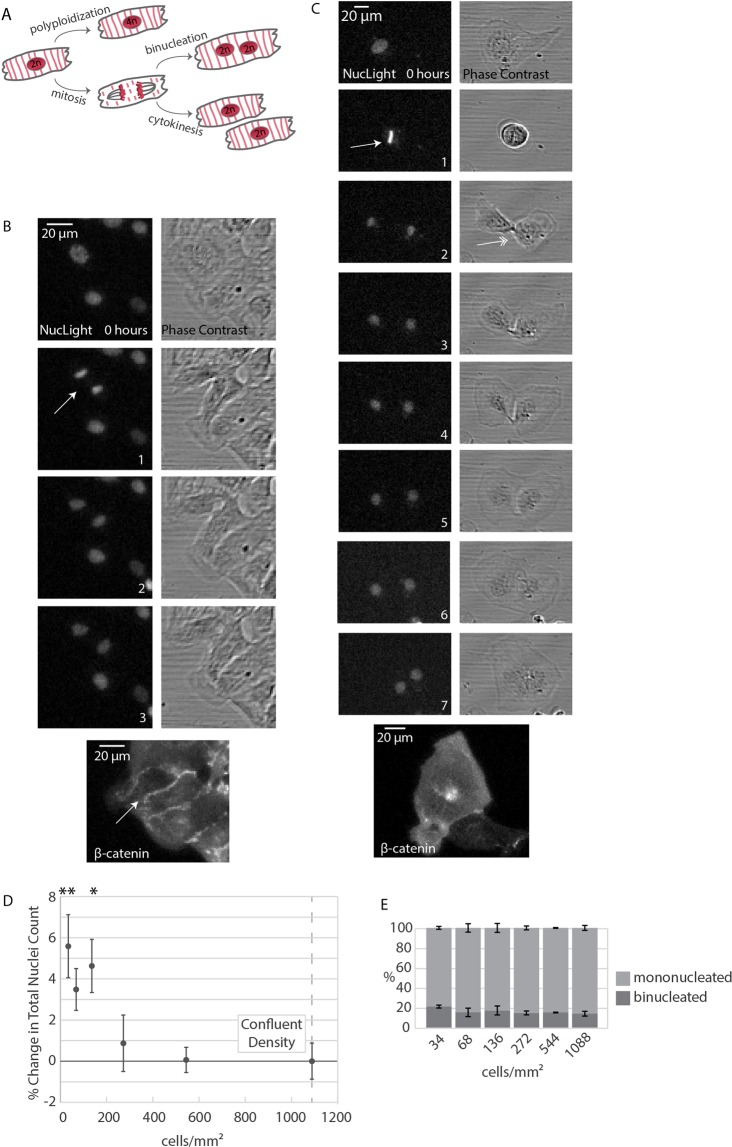
Identifying iPSC-derived human cardiomyocytes as a model for cardiomyocyte proliferation. (**A**) Three possibilities resulting from DNA synthesis: proliferation and polyploidization (i.e., endoreduplication), and two possibilities resulting from mitosis: cytokinesis and binucleation. (**B**) Live montage of dividing hiCM using phase contrast and widefield fluorescence microscopy, visualizing nuclei using a live-cell fluorescent nuclear probe. hiCM were then fixed and stained for β-catenin to confirm division. Arrow: cell-cell boundary. (**C**) Live montage of binucleating hiCM using phase contrast and widefield fluorescence microscopy as in (**B**). Single-headed arrow: metaphase. Double-headed arrow: midbody. (**D**) Proliferative capacity of hiCMs increases with decreasing cell density. Percent change in nuclei count normalized to confluent hiCM so that confluent cells have a 0% change. N = 5 experiments per data point, approximately 500,000 hiCM in total; mean +/− SEM over 5 independent experiments. *p < 0.05. **p < 0.01. (**E**) Binucleation proportion does not change when cells are plated at various densities. Binucleation proportion measured post-fixation stained with β-catenin over three independent experiments. N = 4678 total cells; mean +/− SEM. NS by One-Way ANOVA.

It has been noted that hiCMs have a slight proliferative capacity^5,6^. The vast majority of studies have used methods such as BrdU incorporation into replicating DNA or Ki67 localization in the nucleus to mark cycling cells (Fig. 1). There are two potential outcomes of DNA synthesis: the nucleus becoming polyploid or mitosis. Following mitosis, the cell can either go through cytokinesis and create two new daughter cells (i.e., hyperplastic growth) or it can become binucleated (i.e., hypertrophic growth)^7,8^. While studying sarcomere assembly in hiCMs plated at sub-confluent densities^9^, we observed both hiCMs that appeared to go through cytokinesis (Figs 2B and S1A) and hiCMs which were binucleated (Fig. 2C). We confirmed these two outcomes by localizing β-catenin to mark the boundaries of cells (Figs 2B,C and S1D). We first attempted to use a live-cell membrane marker to identify division events versus binucleation events, but due to the high membrane turnover in cardiomyocytes, live-cell membrane markers immediately become cytoplasmic in localization. While this marker defined boundaries of HeLa cells, the boundaries of hiCMs cannot be defined (Fig. S1B,C). For this reason, we turned to fixed-cell imaging with β-catenin. By marking the boundaries of cells, we can identify if a cell has one or two nuclei.

Interestingly, we found it was rare to detect mitotic events in hiCM plated at high densities, such as a monolayer. Density-dependent proliferation is well documented in other cell types^10^. Indeed, a previous study has shown that human embryonic stem cell-derived cardiomyocytes incorporate more BrdU when plated at “low” density (~268 cells/mm^2^) as opposed to “high” density (~3846 cells/mm^2^)^11^. Given our observations and this previous study, we sought to quantify the relationship between plating density and proliferation in hiCM.

We plated hiCMs at various densities to test if there was a relationship between cell density and proliferation (i.e., mitotic events). In order to get a direct measurement of proliferation, we turned to live-cell microscopy. Every nucleus was labeled with a live-cell nuclear marker, and each well was imaged every 12 hours for a week, followed by quantification to determine the proliferative capacity of the cells. Image stitching was used to visualize the entire population of nuclei in each well of a 96-well plate. This resulted in the monitoring of nuclei in approximately 492,188 hiCMs over the course of 5 independent experiments. As expected, we found an inverse relationship between cell density and proliferation from a range of 544 cells/mm^2^ to 136 cells/mm^2^ (Fig. 2D). Decreasing the density past 136 cells/mm^2^ did not yield corresponding increases in proliferation. 34 cells/mm^2^ was the density at which hiCMs were physically isolated from each other. We next tested whether these observed increases in proliferation were due to binucleation or cytokinesis. Surprisingly, the percentage of binucleated hiCMs was consistent at all densities (Fig. 2E). Taken together, our data suggested that plating hiCMs at sub-confluent densities increases proliferation but does not influence whether a particular hiCM undergoes binucleation or cytokinesis. Based on these results, we next wanted to explore if hiCMs plated at sub-confluent densities could be used for high-content screening to identify modulators of cardiomyocyte proliferation.

### Developing a screen to identify modulators of hiCM proliferation

**Figure 3:**
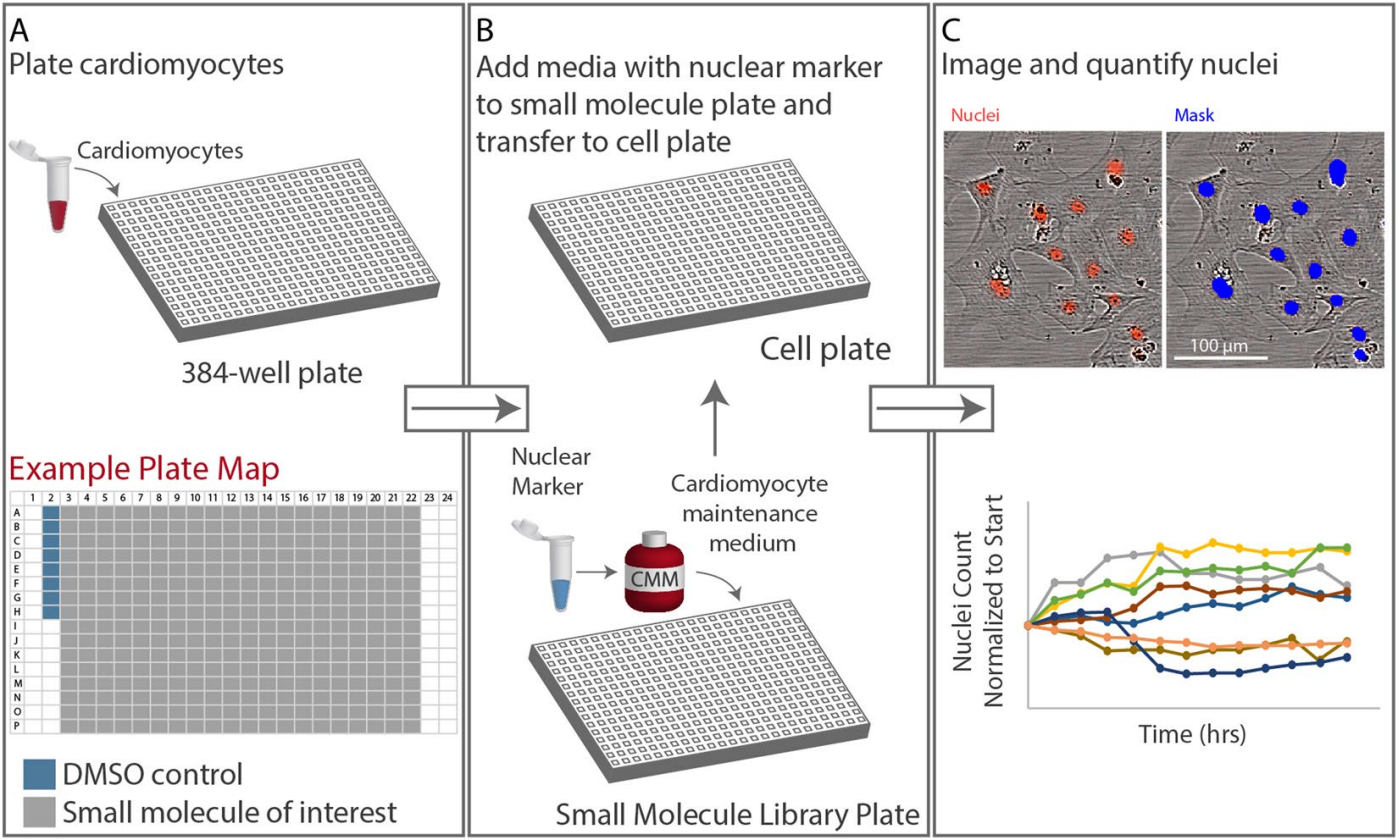
Workflow: identifying small molecule regulators of cardiomyocyte proliferation. (**A**) Cardiomyocyte plating scheme and example plate map. (**B**) Small molecule addition procedure. (**C**) Nuclei imaging and quantification scheme. For more information, see Supplemental Protocol.

We next wanted to identify a plating density that would facilitate detection of both positive and negative modulation of hiCM proliferation, lying in the middle of the inverse relationship between density and proliferation (i.e., 544 cells/mm^2^ to 136 cells/mm^2^). This density would be ~340 cells/mm^2^. Due to the nature of making dilutions of cultured hiCMs, we ultimately chose 333 cells/mm^2^ as our experimental density. To facilitate a high content screen, we also needed to scale-up the number of samples that could be imaged over time. Our experiments presented in Fig. 2 were performed in individual wells of a 96-well plate, however, this can limit the number of molecules that can be tested at once. Thus, we moved to culturing hiCMs in 384-well plates (Fig. 3A). This is cost-effective and allows for the usage of fewer cells per well, and increases the number of possible compounds that can be tested. Of note, cultured hiCMs are fragile and as such are sensitive to physical perturbation during media changes. This rules out less gentle methods of media transfer such as automatic liquid handling, and as such, all media exchanges should be done by hand.

We chose a small molecule library of well-characterized kinase inhibitors from Vanderbilt University’s High Throughput Screening Core. This allows for any results of our screen to be immediately informative as to which signaling pathways are controlling hiCM proliferation. Small molecules were plated into a 384-well plate at 10 mM in DMSO using an automatic liquid handling system. To reduce the concentration to 10 µM, cardiomyocyte maintenance media with a nuclear marker was added to each well of the plate. hiCMs were previously plated into a separate 384-well plate, then the hiCM media was replaced with the small molecule-containing media using a multichannel pipette (Fig. 3B). This allowed for each nucleus in each well to be imaged, masked, and quantified over time (Fig. 3C). We first tested if the nuclear marker we used had any effect on cardiomyocyte proliferation and found no difference in Ki67-positive nuclei in control or labeled cells (Fig. S3C). A detailed protocol is provided in the Supplement.

### Quantifying the effects of small molecules on cardiomyocyte proliferation

**Figure 4:**
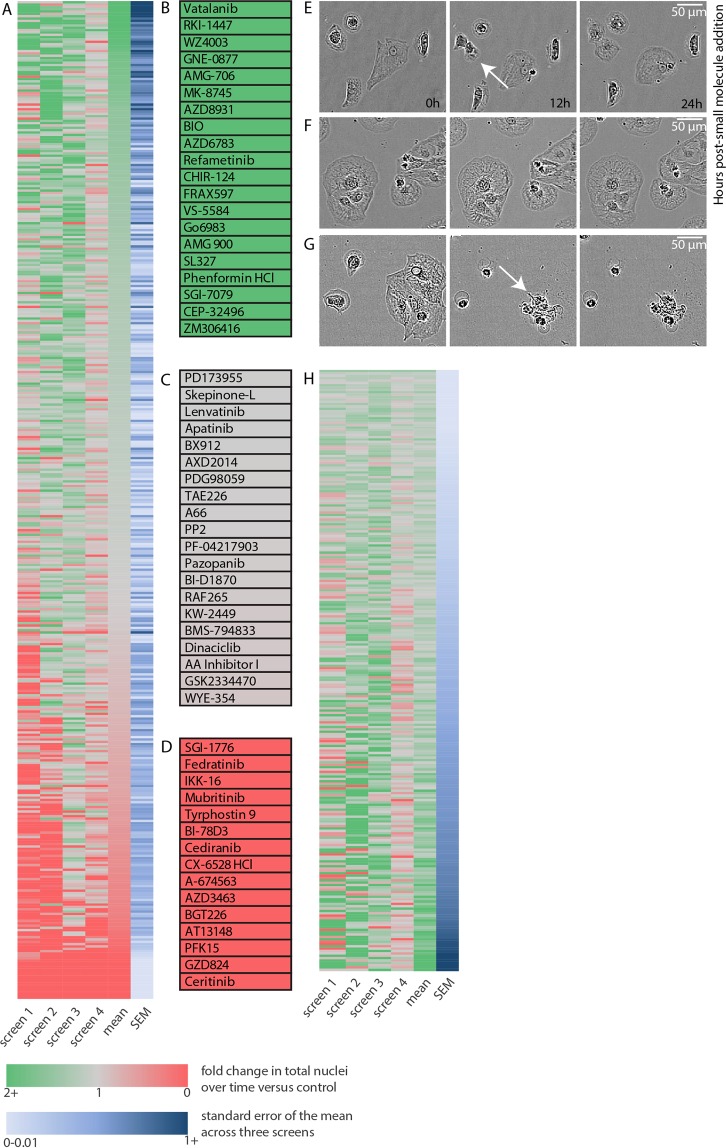
Effect of small molecules on cardiomyocyte proliferation. (**A**) Results of small molecule drug screen on nuclei count over a period of 72 hours, normalized to control, averaged over three independent screens. A value of 1 indicates an equivalent rate of nuclei increase to control cells, that is, there is no apparent change in death or proliferation compared to control. A value of 0 indicates cell death, and a value of 2 indicates a doubling of nuclei count over time compared to control. (**B**) Top small molecules that, on average, increased nuclei count. Note that these calculations do not account for variance between screens. (**C**) Middle small molecules that had no observed effect on nuclei count over time. (**D**) Small molecules that led to 100% cell death reproducibly in each screen. (**E**) Example time-lapse of dividing cardiomyocyte over 24 h post-small molecule addition. (**F**) Example time-lapse of cardiomyocytes over 24 h post-small molecule addition. Note no effect on cardiomyocyte proliferation or death. (**G**) Example time-lapse of cardiomyocytes undergoing cell death after small molecule addition. (**H**) All small molecule compounds with a mean nuclear count increase greater than 1, sorted from top to bottom by smallest to largest standard error of the mean.

Utilizing this protocol, we quantified nuclei count per image over time, taking images every 12 hours for six days. We normalized nuclear increase to control and repeated the screen three more times, resulting in four independent screens using the same small molecules. Interestingly, there was a wide range of changes in hiCM proliferation due to small molecule administration. Some small molecules increased hiCM proliferation, some stopped proliferation altogether, and some caused cell death. The nuclei in each well were quantified as a fold change over time (See equation in the Methods). We averaged the nuclear fold change over four screens (Fig. 4A).

The top small molecules that had the highest average increase in nuclei over time (Fig. 4B), the middle small molecules with no effect on nuclei count (Fig. 4C), and the small molecules that led to death reproducibly in all four screens (Fig. 4D) were identified. We confirmed by manually observing each well (Fig. 4E–G). However, several of these compounds had high standard deviations across screens and did not have a reproducible effect on nuclei count over time, producing false positives. In order to focus on small molecules that reproducibly affected proliferation, we sorted the compounds that had a nuclear fold change greater than 1 by their standard deviation (Fig. 4H). Interestingly, several of these hits have been identified previously as modulators of the cardiomyocyte cell cycle, including GSK-3β inhibitor BIO^12,13^ and a p38-inhibitor SB239063 related to previously identified SB203580^14,15^.

### Phenotypic analysis and small molecule compound follow-up

**Figure 5:**
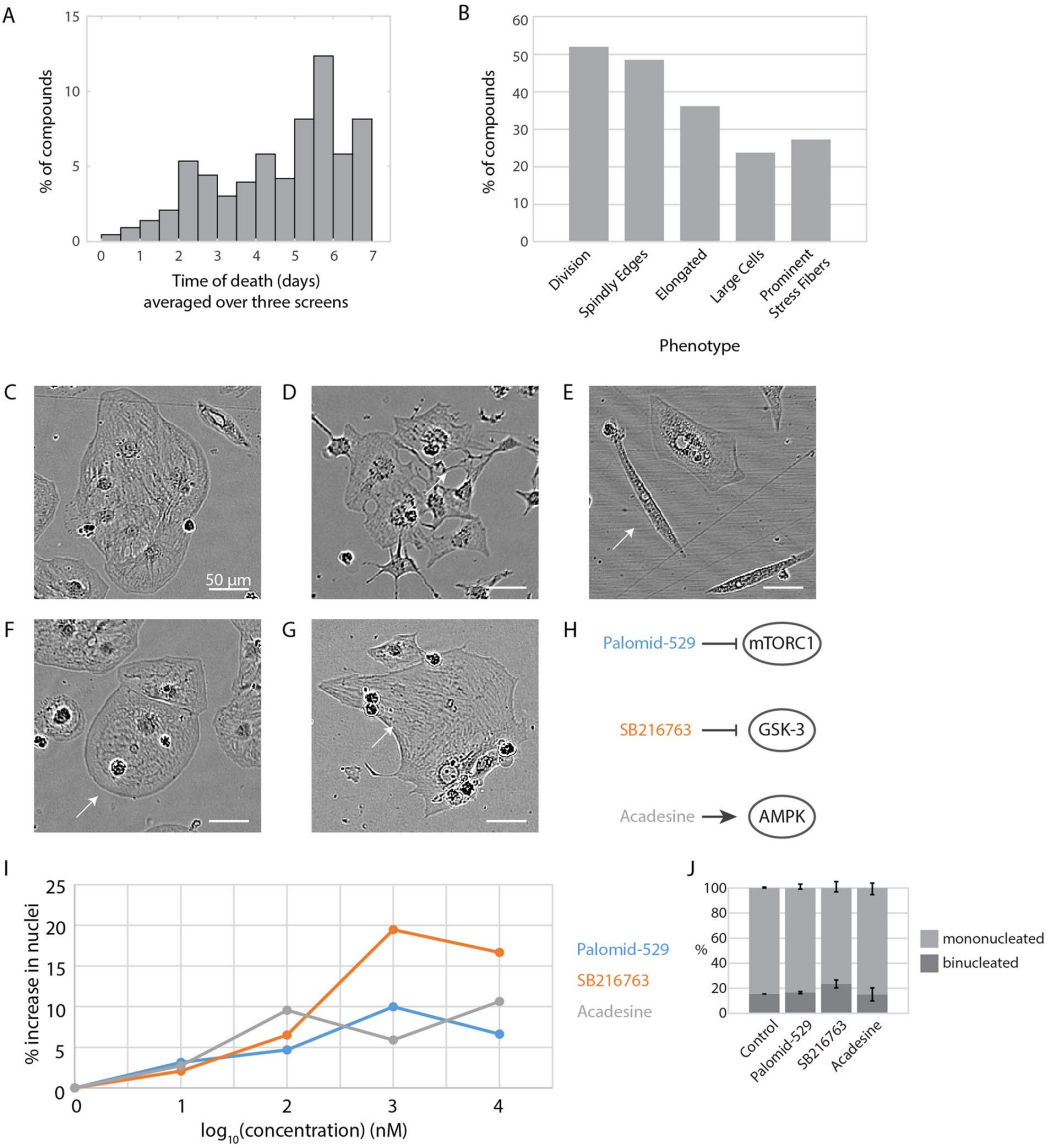
Phenotypic analysis and small molecule compound follow-up. (**A**) Time of death (days) for each well that died within the time frame of the screen, averaged over the three screens. 0 represents that the cells died within the 20 minutes between small molecule addition and initial imaging. 1 represents that cells died before 24 hours post-addition (i.e., between 12 hours and 24 hours). If cells did not die, they are not represented in this graph. Nuclei count was unreliable for cells which died, as there was debris created which fluoresced. Thus, time of death was manually quantified. (**B**) Phenotypic analysis of cardiomyocytes over three screens given 429 small molecules. (**C**) Example of a cluster of approximately 7–8 healthy cardiomyocytes. Small molecule: PD318088. (**D**) Example of a cluster of cardiomyocytes that are especially unhealthy, giving the cells a “spindly” appearance. Small molecule: AT7867. (**E**) Example of an elongated cardiomyocyte (arrow). Note cardiomyocytes directly above the elongated cardiomyocyte that have normal appearances. Small molecule: WAY-600. (**F**) Example of a particularly large cardiomyocyte (arrow). Small molecule: PD318088. (**G**) Example of a cardiomyocyte with stress-fiber like striations on its edge (arrow). Small molecule: APTSTAT3-9R. (**H**) Three small molecules identified by screen as potential regulators of hiCM proliferation. (**I**) Three small molecules from (**H**) tested at 0.01, 0.1, 1, and 10 µM (i.e., 10, 100, 1000, and 10000 nM) over five independent experiments. Shown as mean. (**J**) Binucleation proportion does not change when cells are treated with these small molecules at their most effective concentrations (Palomid-529 5 µM, SB216763 3 µM, and Acadesine 3 µM), measured post-fixation stained with β-catenin. N = 6624 total cells over three independent experiments; mean +/− SEM. NS by One-Way ANOVA.

After the high-throughput screen, we manually observed each well over 7 days and completed a detailed phenotypic analysis. We noted time of death (quantified post-drug addition) for each well that had total cell death within the 7 day timeframe (Fig. 5A). Interestingly, several of the small molecules led to eventual cell death, which further supports that many potential therapeutic molecules might be cardiotoxic. We then noted wells in which there were division events, “spindly” cell edges, elongated cells, particularly large cells, and cells which had prominent stress fibers (Fig. 5B–G, Supplemental Spreadsheet 2).

Once a high-throughput screen is completed specific small molecules need to be identified and further pursued. As an example of such a next step, we chose three small molecules which had high increase in nuclear count over the four screens, but a low variance between screens (Fig. 4H), and that we found of particular interest based on the literature. These three hits included Acadesine, an AMPK activator, Palomid-529, an mTORC inhibitor, and SB216763, a GSK-3 β inhibitor (Fig. 5H). Interestingly, activating AMPK in the heart has been shown to be protective against hypertrophy^16^. In addition, a another small molecule inhibition of GSK-3β using BIO and CHIR99021 has been shown to increase hiCM proliferation^12,13,17^. On average over the four screens, these compounds led to a 20% increase in nuclei count. As we performed our initial screen using small molecules at 10 µM, we next tested the effectiveness of these compounds at lower concentrations. We found that each compound indeed increased proliferation of hiCM at doses lower than the screening concentration (Fig. 5I), and that this induction of proliferation does not affect the baseline balance of cytokinesis and binucleation (Fig. 5J).

### Comparing the number of division events to the overall number of cycling cells

**Figure 6:**
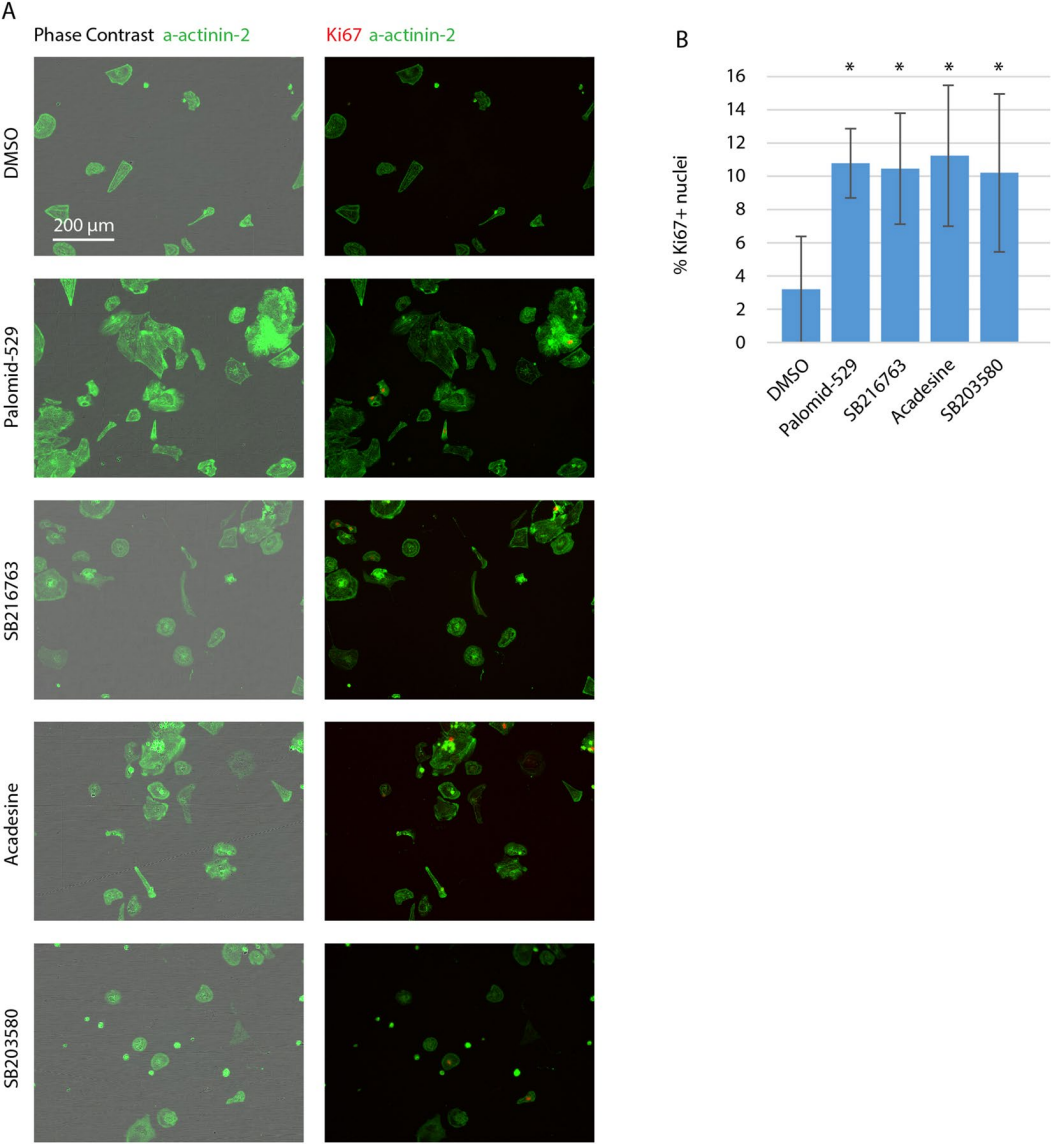
Using Ki67 to confirm three small molecules identified in screen increase hiCM proliferation. (**A**) Phase contrast, α actinin 2 and Ki67 in control hiCM and hiCMs treated with Palomid-529, SB216763, Acadesine, and SB203580. (**B**) Quantification of Ki67+ hiCMs in control and small molecule-treated hiCMs. *p < 0.05.

We next wanted to compare our method to a standard method for identifying cycling cells. We chose the localization of Ki67 using immunofluorescence^18^. We treated hiCMs with the identified small molecules and localized Ki67 and α actinin 2 post-fixation (Fig. 6A). In this way, we can identify cycling cells and simultaneously confirm the cardiomyocyte identity of the hiCMs in control conditions and with the identified small molecules. We also localized Ki67 in hiCMs treated with a previously identified small molecule that has been shown to induce cardiomyocyte proliferation, a p38 inhibitor^14^. Each of the three identified compounds increased the percentage of Ki67-positive nuclei to similar or even higher levels than the previously identified p38 inhibitor (Fig. 6B). However, the identification of cycling cells using Ki67 accounts for more cells than those which actually subsequently divide^19^.

We also wanted to compare the cost of performing a screen using live-cell nuclear localization with the current method of fixed-cell Ki67 localization (Table 1) and found that our method of performing the screen was significantly more cost-effective. We also include a detailed protocol on how to complete both methods of screens, including step-by-step alternative approaches for laboratories with more limited resources with the goal to alleviate the significant financial investment typically required for starting research in the cardiovascular field. In conclusion, fixed-cell localization of cycling markers only allows for imaging one time point and provides an overestimate of cell division, and further, is more costly than live-cell imaging of nuclei.

**Table 1:** Cost per 384 well plate of screening platform.

Reagent	Cost per plate
384-well plate	$5.84
384 small molecules aliquoted by the Vanderbilt HTS core	$40
Cells	$341.33
Media	$18.93
NucLight	$75.84 (two washes)
Ki67	$130

## Discussion

The diversity of the scientific community working on a problem is instrumental to the innovation of any field. Previously, the study of cardiomyocytes has been limited to researchers with substantial funding and specialized training to initiate *in vivo* studies or differentiation protocols from iPSCs. Here we present a method that is specifically designed to be straight-forward and accessible to a wide variety of researchers. hiCMs can be purchased, which bypasses the typically rate-limiting and costly step of initiating in-house hiCM-differentiation. Furthermore, the experiments do not require advanced microscopy. Indeed, all the experiments in the main figures were completed using relatively low magnification imaging with a 4x, 10x, or 20x objective, though one objective would be sufficient. Indeed, we have provided a detailed protocol in the supplement that presents how our method can be modified to accommodate any microscope capable of performing fluorescence. Using our method, we present several datasets as a resource for determining small molecules that play an activating or inhibitory role in cardiomyocyte proliferation. Screening a limited compound library revealed many potential regulators of proliferation and cell death (Supplemental Spreadsheets 1, 3). We also provide additional cellular phenotype information for each compound (Supplemental Spreadsheet 2).

We chose to use purchased hiCMs because the initial investment is far less than in-house differentiation protocols. In-house hiCM differentiation protocols also do not produce pure populations of myocytes. These protocols can contain ~10% of nonmuscle cells^20^. Because the percentage of dividing hiCMs is so low, a small contamination of highly proliferative non-muscle cells, e.g., fibroblasts, could skew the results. Each batch of purchased cardiomyocytes comes with a certificate of purity, claiming >99%. However, even if 0.99% of the cells were highly proliferative, this would affect results. For this reason, we routinely localize α actinin 2 in our cultures. These cells are positive for α actinin 2. Of note, over the course of this study, we have only found 3 cells present in our cultures that do not express α actinin 2 out of the ~500,000 we have observed. Further, every dividing cell found in cultures stained for α actinin 2 has been α actinin 2-positive (Fig. 6A and S1A). Taken together, these cultures contain cardiomyocytes which have the capacity to divide.

Another factor to consider when using hiCMs for any experimental purpose is their ‘maturity’. It has been well-documented that hiCMs do not resemble *in vivo* adult human cardiomyocytes transcriptionally or morphologically^21^. In addition, adult human cardiomyocytes have a very low potential for division^5,6^. Conversely, hiCMs transcriptionally resemble fetal/neonatal cardiomyocytes^21^, and obviously can divide. It is for this reason that we believe hiCMs are an ideal system to explore what regulates the cell cycle and cytokinesis in cardiomyocytes.

There are several reasons we chose to count nuclei as the basis of this screening protocol, rather than previously utilized methods. Proliferative markers such as Ki67 and PCNA give an overestimate of cell proliferation, as they mark any cell that is cycling in general. This can mark cells undergoing endoreduplication (i.e., polyploidization), cytokinesis, and binucleation. DNA synthesis markers such as BrdU/EdU detects these three outcomes as well as DNA damage repair^19^. To circumvent these issues, researchers have turned to midbody markers such as Aurora B kinase to more directly mark cytokinesis. However, groups have recently shown preliminary evidence that binucleating cells can form a midbody^22,23^. Interestingly, we also see examples of binucleating cells that have a clear midbody in a typical position (Fig. 2C, double-headed arrow). Thus, using midbody markers or even simply identifying mitotic figures does not unequivocally mark cardiomyocytes undergoing cytokinesis. Hesse & Doengi^22^ also propose using distance between nuclei after mitosis to differentiate between cytokinesis and binucleation. However, some hiCMs have two nuclei that are well-separated (Supplemental Fig. 2F).

An *in vivo* approach to navigating cardiomyocyte proliferation is to use the MHC promoter to mark only cardiomyocytes and use sophisticated tools such as mosaic analysis using double markers (MADM), etc., to measure CM proliferation^2^. However, the MHC promoter could be active in undifferentiated progenitor cells^19^ which could cloud results in mouse models. In fixed tissue, thin sections restrict the identification of binucleated cardiomyocytes. In thicker sections, optical aberrations, limited axial resolution, and imprecise marking of cell boundaries are limitations. Even so, *in vivo* approaches limit the throughput of identifying any compounds or factors that affect cardiomyocyte proliferation.

Given the ambiguity that results from other markers, we propose using live hiCMs for high-throughput screening of regulators of hiCM proliferation by simply counting nuclei. This method allows for a more accurate proliferation approximation. We present this dataset as a resource for other cardiac researchers to follow up on any small molecule or phenomenon of interest. With a nearly endless variety of live-cell markers available and the ease of this protocol, it can be adapted to study several aspects of cardiovascular cell biology. Here we used a small molecule library as a proof-of-concept; however, CRISPR-based screens can be easily incorporated into this platform. Such a simplistic experimental platform can bridge the gap between a limited screen providing preliminary data and a hypothesis-driven follow-up study. hiCMs have been shown to be amenable to genetic and pharmacological perturbations^3,9^. Indeed, we have provided suggestions in the supplemental protocol as to when genetic or pharmacological experiments can be incorporated.

## Supplementary information

Supplementary Information

Detailed Drug Info

All Screen Notes

Screen Quantification
