# Supplementary material for: A simple and flexible high-throughput method for the study of cardiomyocyte proliferation
Source: Sci Rep. 2019 Nov 4;9:15917. doi: 10.1038/s41598-019-52467-0 (PMC6828730; doi:10.1038/s41598-019-52467-0)
Supplement: Supplementary file 1 — Supplementary Information [file 41598_2019_52467_MOESM1_ESM.pdf]

A simple and flexible high-throughput method for the study of cardiomyocyte proliferation

Abigail C. Neininger[1], J. Hunter Long[1], Sophie M. Baillargeon[1], & Dylan T. Burnette[1, 2]

[1]Department of Cell and Developmental Biology, Vanderbilt University, Nashville, TN, United States. 37232

[2] To whom correspondence should be addressed: dylan.burnette@vanderbilt.edu 1161 21st Ave S, Medical Center North Room T2209, Nashville, TN. 37232.

SUPPLEMENTAL MATERIAL

KEY RESOURCES

REAGENT or RESOURCE	SOURCE	IDENTIFIER
**Antibodies**		
Rabbit-anti-NMIIB	Cell Signaling Technology	8524S Clone: D8H8
Mouse-anti-α-actinin-2	Sigma	A7811 Clone: EA53
Rabbit-anti-NMIIA	BioLegends	909801 Clone: Poly19098
Mouse-anti-α-tubulin	Sigma	B512 Clone: DM1α
Mouse anti-β-catenin	BD Biosciences	610153
Rabbit-anti-Ki67	Cell Signaling Technology	9129 Clone: D3B5
Rabbit-anti-pH3	Cell Signaling Technology	9701
Mouse-anti-BrdU	Cell Signaling Technology	5292
Goat anti-mouse 488	LifeTechnologies	A11001
Goat anti-rabbit 488	LifeTechnologies	A11034
Goat anti-mouse 568	LifeTechnologies	A11004
Goat anti-rabbit 568	LifeTechnologies	A11036
**Biological Samples**		
Cardiomyocytes[2]	Cellular Dynamics	CMC-100-012-000.5
Bovine Serum Albumin	RPI	A30075-100.0
**Chemicals, Peptides, and Recombinant Proteins**		
NucLight Rapid Red	Essen Biosciences	4717
Vectashield with DAPI	Vector	H-1200
CellBrite Fix Membrane Stain	Biotium	30088, 30090
Cardiomyocyte Plating Medium	Cellular Dynamics	M1001
Cardiomyocyte maintenance medium	Cellular Dynamics	M1003
Kinase Inhibitor Library	Selleck Chemicals	L1200
Acadesine	Selleck Chemicals	S1802
SB216763	Selleck Chemicals	S1075
SB203580	Selleck Chemicals	S1076
Palomid-529	Selleck Chemicals	S2238
PBS, 10X, Ca^{2+}/Mg^{2+} free	Gibco	70011-044
Paraformaldehyde, 16%	Electron Microscopy Sciences	15710
PBS, 10X, with Ca^{2+}/Mg^{2+}	Corning	46-013-CM
0.5% Trypsin	Gibco	15400-054
0.1% Gelatin	Sigma	ES-006-B
Dimethyl Sulfoxide	Sigma	276855
Triton X-100	Fisher Scientific	BP151100
CellLight Nucleus	Invitrogen	C10602
BrdU	Abcam	Ab142567
**Software and Algorithms**		
MATLAB 2017b	MathWorks	
FIJI/ImageJ	NIH, Open Source	

MATERIALS AND METHODS

Cell culture and chemicals

iPSC-derived cardiomyocytes (hiCM, CMC-100-012-000.5, Cellular Dynamics, Madison, WI) were cultured in polystyrene 96-well plates (3599, Corning, Corning, NY) or 384-well plates (781182, Greiner, Kremsmünster, Austria or 164688, ThermoFisher, Waltham, MA) coated with gelatin (ES-006-B, Sigma, St. Louis, MO) in growth medium (M1003, Cellular Dynamics, Madison, WI) as per manufacturer's instructions. Cells were cultured at 37°C and 5% CO_2. For re-plating hiCMs onto glass substrates (Figure S1A-D), cells were washed 2x with 100 µL 1x PBS with no Ca^{2+}/Mg^{2+} (PBS*, 70011-044, Gibco, Grand Island, NY). PBS* was completely removed from hiCMs and 40 µL 0.1% Trypsin-EDTA with no phenol red (15400-054, Gibco, Grand Island, NY) was added to hCMs and placed at 37°C for 2 minutes. Following incubation, the culture well was washed 3x with trypsin inside well, rotated 180 degrees, and washed another 3x. Trypsinization was then quenched by adding 160 µL of culture media and total cell mixture was placed into a 1.5 mL Eppendorf tube. Cells were centrifuged at 1000xg for 3 minutes, and the supernatant was aspirated. Cells were then re-suspended in 200 µL of culture media and plated on 35 mm dishes with 10 mm glass bottom (D35-10-1.5-N; CellVis, Sunnydale, CA) pre-coated with 10 µg/mL fibronectin (#354008, Corning, Corning, NY) for 1 hr at 37°C. Reagent reservoirs were purchased from VWR (89094-682, Radnor, PA).

Mouse anti-β-catenin (1:200, 610153) was purchased from BD Biosciences (San Jose, CA). Mouse anti-α-actinin-2 (1:200, A7811, clone EA-53) and mouse anti-α-tubulin (1:200, B512, Clone DM1α) were purchased from Sigma (St. Louis, MO). Rabbit anti-NMIIA (1:1000, 909801 Clone Poly19098) was purchased from BioLegends (San Diego, CA). Rabbit anti-NMIIB (1:200, 8524S), rabbit anti-Ki67 (1:200, 9129), mouse anti-BrdU (1:200, 5292S), and rabbit anti-pH3 (1:200, 9701S) were purchased from Cell Signaling Technologies (Danvers, MA).

Alexa Fluor 488-goat anti-mouse (A11029), Alexa Fluor 488-goat anti-rabbit (A11034), Alexa Fluor 568-goat-anti-rabbit (A11011), and Alexa Fluor 568-goat anti-mouse (A11004) antibodies were purchased from Life Technologies (Grand Island, NY).

BrdU (ab142567, abcam, Cambridge, UK) was reconstituted to 10 mM in water, then to 10 µM in cardiomyocyte maintenance medium and filtered through a 0.22 µm syringe filter. Cells were incubated with 5 µM of each follow-up compound and BrdU for 20 hours, then fixed and permeabilized as below (see "Fixation and immunostaining"). Then, to hydrolyze DNA, cells were incubated with 1 M HCl for an hour at room temperature, then neutralized with 0.1 M sodium borate buffer (pH 8.5) for 20 minutes at room temperature. Next, cells were washed with PBS and immunostaining continued as usual (see below).

Small molecule kinase inhibitor library preparation and screen

Briefly, a small molecule kinase inhibitor library was acquired from the High Throughput Screening Core at Vanderbilt University, which was originally purchased from Selleck Chemicals (L1200, Houston, TX). 50 nL of each compound was placed in wells of a 384-well plate using an Echo 555 Liquid Handler (Labcyte Inc., San Jose, CA) at 10 mM in DMSO (276855, Sigma, St. Louis), and were subsequently resuspended in 50 µL of cardiomyocyte maintenance media to a final concentration of 10 µM. Four days post-thaw, the cell media was replaced with media containing the compounds of interest and 1:4000 NucLight Rapid Red Reagent (4717, Essen Biosciences, Ann Arbor, MI). This step was repeated again three days later. All control wells contained an equal volume of DMSO: 50 nL. An in-depth protocol can be found below.

Fixation and immunostaining

Cells were fixed with 4% paraformaldehyde (PFA, 15710, Electron Microscopy Sciences, Hatfield, PA) in PBS (46-013-CM, Corning, Corning, NY) at room temperature for 20 min and then extracted for 5 min with 1% Triton X-100 (BP151100, Fisher Scientific, Suwanee, GA) and 4% PFA in PBS as previously described[69]. For immunofluorescence experiments, cells were blocked in 5% bovine serum albumin (BSA, 30075-100.0, RPI, Mount Prospect, IL or A3059, Sigma, St. Louis, MO) in PBS, followed by antibody incubations. VectaShield with DAPI (H-1200, Vector Laboratories Inc., Burlingame, CA) was used for mounting.

Structured Illumination Microscopy

SIM imaging and processing (Figure S1A) was performed on a GE Healthcare DeltaVision OMX equipped with a 60x 1.42 NA oil objective and sCMOS camera.

Fluorescence and live-cell microscopy

Some wide-field fluorescence images (Figures S1B-D) were acquired on a Nikon Eclipse Ti equipped with a Nikon 20x 0.4 NA air objective and a Nikon DS-Qi2 CMOS camera. All other images were acquired on an Incucyte (4647, Essen Biosciences, Ann Arbor, MI) at 4x, 10x, or 20x, at 37°C and 5% CO_2.

Data Quantification

For nuclei count over time (Figures 2D, 4A-D, H, 5I), nuclei were counted using the IncuCyte analysis interface. Briefly, nuclei were thresholded using rolling ball background subtraction for each time point. Data were normalized to controls, normalizing control wells to a 1 fold-change or a 0% nuclear count increase. All values shown are mean +/- SEM over three experiments. Binucleation was manually quantified (Figures 2E, 5J, S1F) using an antibody for β-catenin and a nuclear marker (NucLight, 4717, Essen Biosciences, Ann Arbor, MI). Cells on the edge of a field of view that had a portion cut off were not included. Ki67-positive percentage was calculated as percentage of nuclei which were positive for Ki67 localization.

Statistics

P-values were calculated using One-Way ANOVAs with the exception of Figure S3F. In Figure 2D, 2E, 5J, repeated measures One-Way ANOVAs were performed. For Figure 5J, a one-way ANOVA without repeated measures was performed. A Dunnett's post-hoc test was performed if the ANOVA p-value was less than 0.05. In Figure S3F, a two-tailed paired student's t-test was performed.

DETAILED PROTOCOL

We used a relatively small library of 429 small molecules for this proof of concept manuscript. In total, we used 445 wells of a 384 well plate (Figure S1G). This included the library with 16 accompanying DMSO controls. As such, we needed 1.5 million human iPSC-derived cardiomyocytes (hiCM) to plate at a density of ~333 hiCM/mm^2, that is, 3333 hiCM/well; accounting for pipette variance. This density was determined by calculating the middle density between the densities at which the hiCMs proliferate the least and the most (Figure 1D).

Thawing Cardiomyocytes: Day 1

> Note: Follow manufacturer's instructions and/or a differentiation protocol[68] to obtain 1.5 million cardiomyocytes. We used a variation on Cellular Dynamics plating protocol for a 500 µL tube containing approximately 3 million cells. We thawed the hiCMs into wells of a 384-well plate. This plate was used directly for the experiments as we noticed that re-plating hiCMs caused loss of hiCMs due to cell death. We allowed at least one row of empty wells on the sides of the plate. This minimizes contamination and evaporation, which is an important concern when media is not being changed daily.

> Note: There are two main companies that provide quality differentiated hiCMs that we have used. We chose Cellular Dynamics because that is what we had frozen in our liquid nitrogen storage at the start of this project. Cellular Dynamics hiCMs are frozen at 30 days of differentiation. We have also used hiCMs from Ncardia to study sarcomere assembly and found them to be similar to those from Cellular Dynamics[70]. However, we have not hiCM for cell from Ncardia for proliferation studies as of yet.

- Thaw plating medium at room temperature overnight and thaw maintenance medium at 4°C overnight.
- 2 hours before thaw, add 25 µL 0.1% sterile gelatin (ES-006-B, Sigma, St. Louis, MO) to each well of a 384-well plate in a cell culture hood and incubate at 37°C for 2 hours. We use our cell culture incubator for all steps requiring 37ºC, unless otherwise noted.
- Immediately before the thaw, aspirate gelatin from all 445 wells in a culture hood and return to 37°C.
- Remove a vial of cells from the vapor phase of liquid nitrogen storage using a large pair of forceps and warm the tube in a 37°C water bath for 3 minutes. Take care to maintain sterility by not submerging the cap of the tube, and hold the tube stationary- NO SWIRLING. The large pair of forceps may be used to hold to tube in the water bath, or a floating tube rack can also be used as long as the cap remains above water.

- Remove the cryovial from the water bath, spray it with 70% ethanol, and place it into the culture hood.

> Note: It is important to optimize which plate to use based on the type of image information your experiment requires (e.g., fluorescence and/or phase contrast). For example, Greiner 384-well plates (781182) a have larger font size on the plates, allowing for easy addition of small molecules, and work well for fluorescent imaging as they have no detectable auto-fluorescence (Figure 2C). However, these plates produce an optical aberration that creates linear patterns, and often have small scratches (Figure 2C). On the other hand, some plates (e.g., Thermo Fisher 384-well plates- 164688) do not produce such patterns and have less. However, these plates are labeled with a smaller font that is difficult to read from afar when the plates are in a laminar flow hood behind glass. 96-well plates or even fewer/bigger wells may be used as well. This increases sample size at the expense of cost and overall throughput.

- In the cell culture hood, use a 1 mL pipette to slowly remove cells from the cryovial over two seconds and them to a sterile 50 mL conical centrifuge tube (89039-656, VWR International, Suwanee, GA or 82050-348, Greiner, Kremsmünster, Austria) by slowly expelling them over 4 seconds.
- Gently rinse empty cryovial with 1 mL plating medium to recover remaining cells. To do so, tilt the pipette tip so that the media runs down the inner sides of the tube, rotating the pipette tip to rinse the circumference of the tube. Transfer the 1 mL of plating medium with the recovered cells from the cryovial to the 50 mL conical tube drop-wise over 90 seconds while gently swirling the conical tube. Slow addition of medium in this step is critical to minimizing osmotic shock: add approximately one drop per ~3 seconds.
- Slowly add 4.5 mL of plating medium to the 50 mL conical tube. Add each remaining 1 mL of medium drop-wise over 30 seconds per mL. Gently swirl the tube while adding medium.
- Close the cap on the 50 mL conical tube and invert gently 2 times. Do not shake or vortex the cell suspension.

> Note: To determine how much plating medium to add, see cell viability calculation of the batch of hiCM purchased. Each 50,000 cells will require a total of 100 µL of medium. For example, a batch with 3 million viable cells will produce 60 x 50,000 cells, so 60 x 100 µL = 6 mL is required in total. After the 1.5 mL (500 µL cells + 1 mL plating medium used to wash cryovial), 4.5 mL remains.

- Transfer 3 mL of the cell suspension to a new sterile 50 mL conical tube. The remainder of the cell suspension may be used for other purposes such as second screen or other experiments.
- Add 19.5 mL of plating medium to the 3 mL of cell suspension, cap the tube, and invert gently 2 times.
- Pour the entire contents of tube (~22.5 mL) into a sterile reagent reservoir.
- Retrieve the two 384-well plates from incubator and transfer to the culture hood.
- Use the multichannel pipette set to 50 µL to add cells from the reservoir into the wells of the 384-well plates, using the plate map of choice.
- Put the plates into the incubator for at least 5 hours (Note: we have gone up to 7 hours). This allows time for the cells to recover and adhere to the plate so that they are not washed away during the media change.
- During the incubation in Step 15, warm 22.5 mL of maintenance medium to 37°C in a bead bath, water both or incubator.
- 5-7 hours post-thaw, retrieve the plates from incubator and put them in the cell culture hood.
- Pour the warm maintenance medium into new sterile reagent reservoir in the cell culture hood.
- With two multichannel pipettes set to 50 µL each, simultaneously remove 50 µL of plating medium from the wells with one hand and add 50 µL of maintenance media from reservoir to the cells with the other hand.

> Note: Be careful not to touch the bottom of the wells while changing media, and minimize time the cells are without media. These cells are fragile, so all media changes in this protocol are done by hand rather than with a liquid handling system. A makeshift waste container for large quantities of pipette tips can be made out of a tip box lid. If two multichannel pipettes are not available, add new media immediately after removing old media, row-by-row.

- Put the cells back in the incubator for 2 days.

Maintaining cardiomyocytes: Day 3

21. Two days post-plating, change the media as in step 19. Warm 22.5 mL of media. Remove 50 µL from each well and add 50 µL simultaneously from reagent reservoir, being careful not to touch or disrupt cells.

> Note: If transducing a stably-expressing nuclear marker (or transducing or transfecting any other construct), it is possible to do so today. For example, if using Cell Light Nucleus (C10602, Invitrogen, Carlsbad, CA).

Small molecule addition: Day 5

22. Warm 22.5 mL of maintenance medium to 37°C in bead bath, water bath or incubator.

23. Add the nuclear marker to maintenance medium at desired concentration
    a. We used NucLight Rapid Red at a 1:4000 dilution (i.e., 5.6 µL NucLight to 22.5 mL medium).
24. Pour ~1/2 of the contents of the conical tube containing media into a sterile reagent reservoir in hood, then close the conical and return it to the bead bath or equivalent to maintain temperature and sterility. (Small molecule addition can take several minutes depending on the researcher's pipetting speed, and medium that cools down will be dangerous to the cells)

> Note: For our purposes, we obtained two 384-well plates with plate maps as in Figure S1G from the Vanderbilt University High Throughput Screening Core, with 50 nL of each small molecule from the SelleckChem Kinase Inhibitor Library per well at 10 mM. The plates were sealed and stored for ~6 hours in a desiccator until use. In this way, it is simple to add media to each well of the 'small molecule plate', then transfer media from the small molecule plate to the cell plate, 12 wells at a time using a multichannel pipette. We recommend having the small molecules of interest in a separate plate matching the planned map of the cell plate in order to simplify small molecule addition and minimize contamination. See plate maps in Figure S1G or troubleshooting for more information

25. Remove cell plate 1 from the incubator and remove seal from small molecule plate 1 in the hood.
26. Pipette the small molecules in this order (see troubleshooting for addition details on the handling procedure):

a. Add 50 µL of media to the small molecule plate using a multichannel pipettor and gently mix by pipetting up and down (this dilutes the small molecules to 10 µM).

b. Remove 50 µL of media from the corresponding wells of the cell plate using a second multichannel pipette.

c. Transfer 50 µL media from the small molecule plate to the corresponding wells of the cell plate using first multichannel pipette. Discard both sets of pipette tips.

Note: We used an automatic microscope that can image up to 6 plates at a time. However, any microscope with an automatic stage and multipoint capabilities could be used. This would limit the number of samples imaged at a time as most microscopes can only image one plate at a time. Alternatively, if live-cell imaging is not available, fixed time points at various hours or days post-small molecule addition and imaging of nuclei with a marker such as DAPI will also facilitate nuclei count.

27. Repeat Step 26 for plate 2, adding warm media to the reagent reservoir when needed. Take care to avoid cross-contamination by replacing tips at every step. For 445 wells, this protocol will use ~9 pipette tip boxes per drug addition day.

28. Place the cell plates on the microscope of choice and start imaging.

Small molecule addition (Day 8: Repeat Steps 22-28)

29. Warm 22.5 mL of the maintenance medium to 37°C in a bead bath or equivalent.

30. Obtain the new plate of small molecules as previously.

31. Similarly add media to the small molecule plate, remove media from the cell plate, and transfer media from the small molecule plate to the cell plate as previously (Steps 22-28).

32. Place cell plates back on the microscope.

Quantify (Day 11)

33. Remove cells from automatic imager 6 days post-small molecule addition 1.
34. Quantitative assessment of cardiomyocyte proliferation. We used the Incucyte automatic analysis interface to do so, but thresholding nuclei in FIJI/ImageJ will also provide a mask to quantify nuclei count over time.

Fix and stain cells (Optional- Day 11)

35. Prepare 4% Paraformaldehyde (PFA) in PBS in a chemical fume hood. Prepare 25 µL per well.
    a. Sample calculation for 445 wells: Prepare 12 mL of 4% PFA by adding 3 mL 16% PFA to 9 mL 1X PBS.
36. Gently aspirate media from each well of the cell plate and add 25 µL 4% PFA immediately afterwards. For this step and following steps, minimize amount of time cells are dry.
37. Wait 20 minutes.
38. Aspirate each well and wash by adding 25 µL PBS.
39. Repeat wash step (Step 38) twice more.
40. Prepare 4% PFA, 1% Triton X-100 permeabilization solution in a chemical fume hood, 25 µL per well.
    a. Sample calculation for 445 wells: Prepare ~12 mL of permeabilization solution by adding 3 mL 16% PFA and 120 µL Triton X-100 to 9 mL 1X PBS.
41. Aspirate PBS from each well of the cell plate and add 25 µL of the permeabilization solution to each well immediately afterwards.
42. Wait 5 minutes.
43. Wash the wells 3X each with 1X PBS as in Steps 38-39.
44. Prepare 5% BSA blocking solution in 1X PBS (25 µL per well).
    a. Sample calculation: Add 2.5 grams BSA powder in a 50 mL conical tube, fill the tube to 50 mL with 1X PBS and vortex until the powder is dissolved.
45. Aspirate the PBS from each well of the cell plate and add 25 µL 5% BSA in PBS to each well immediately afterwards.
46. Wait 20 minutes.
47. Prepare primary antibodies to the desired dilution.
    a. Sample calculation for 445 wells, staining for β-catenin (610153, BD Biosciences, San Jose, CA) and a rabbit antibody of choice, both at 1:200: Add 34 µL antibody to 6.8 mL 5% BSA.

> Note: We recognize this uses a large quantity of antibody, which can be costly. To circumvent this issue, we recommend fixing all wells and keeping the plates in a 4°C fridge until the wells are quantified and any hits are identified. Then, only the relevant wells can be stained to conserve antibody. 15 µL is also the minimum requirement for these steps: most 384-well plate wells can hold up to 100 µL of liquid.

48. Aspirate the BSA from each well of the cell plate and add 15 µL of primary antibody to each well.
49. Wait 1 hour 45 minutes.
50. Prepare the secondary antibodies to the desired dilution.
    a. Sample calculation for 445 wells, using 488-goat-anti-rabbit and 568-goat-anti-mouse at 1:100: Add 68 µL of each secondary antibody to one tube containing 6.8 mL 5% BSA.
51. Centrifuge secondary tube at 10,000xg for 2 min to pellet aggregates.
52. Meanwhile, wash each well with 5% BSA as in Steps 38-39.
53. Aspirate the BSA out of each well and add 15 µL of secondary to each well immediately after.
54. Wait 1 hour.
55. Wash each well with 1X PBS as in Steps 38-39.
56. Keep 25 µL of PBS remaining in each well.
57. Put plates on the microscope and image immediately to minimize exposure to 37°C.

> Note: If using a 96-well plate, double the calculations, i.e. wash with 50-100 µL PBS or BSA and add at least 30 µL of antibody per well.

TROUBLESHOOTING

- Detailed procedure for small molecule addition: (All steps should be done in a cell culture hood)
    - Add 50 µL of maintenance media from a sterile reagent reservoir to the small molecule plate with right hand, using a multichannel pipette.
    - Pipette the media in the small molecule plate up and down gently with the right hand to mix the media with the small molecule. Do not discard the right hand pipette tips yet.
    - With left hand, remove 50 µL of media from the corresponding wells of the cell plate. Do not touch the bottom or scrape the cells.
    - Discard the left hand pipette tips with the media into waste container 1.
    - With right hand, pull up 50 µL of media from the small molecule plate wells from Step 1.

  - With right hand, add the 50 µL from the small molecule plate to the wells of the cell plate that had media removed in Step 3.
  - Discard the right hand pipette tips into waste container 2.
  - Repeat Steps 1-7 for the entire plate.

TIMING

Total with purchased myocytes: 11 days

- Steps 1-20 (Day 1): 1 day (~6 hours)
- Step 21 (Day 3): 1 day (~1 hour)
- Steps 22-28 (Day 5): 1 day (~2 hours)
- Steps 29-32 (Day 8): 1 day (~2 hours)
- Steps 33-34 (Day 11+): 1 day (~2 hours)
- Steps 35-57 (Day 11+): 1 day (~5 hours)

Total with in-house differentiated myocytes: 42 days

- Skip steps 1-21
- Maintenance of hiPSCs (Days 1-7): 1 week[68]
- Differentiation of hiPSCs (Days 8-21): 2 weeks[68]
- Re-plating and growing hiCMs for maturation (Days 22-35): 2 weeks[68]
- Steps 22-28 (Day 36): 1 day (~2 hours)
- Steps 29-32 (Day 39): 1 day (~2 hours)
- Steps 33-34 (Day 42+): 1 day (~2 hours)
- Steps 35-57 (Day 42+): 1 day (~5 hours)

SUPPLEMENTAL EXCEL FILES

“AllScreenNotes.xlsx”

This file shows the detailed phenotypic observations for each well of four screens. Each sheet of the excel file shows the well of the plate, the identifier of the small molecule, and phenotypic information, including when the majority of the cells in the well died and their appearance. Each observation was done blinded to the small molecule identification.

“ScreenQuantification.xlsx”

This file shows the raw data for the calculations in Figure 3. The first sheet shows the data for Figure 3A, that is, all of the data for each screen sorted by the average (Columns A-F). The first sheet also shows all wells with a nuclear fold change greater than one sorted by the standard error of the mean (Columns I-N). The second through fifth sheets show the raw nuclear counts for each well in the first, second, and third screens, respectively. Note that the first screen had a different plate map than the rest, and that the conversion between screens can be found in the first sheet of “AllScreenNotes.xlsx”. Screens 3 and 4 used a different nuclear marker than screens 1 and 2, so the nuclear fold changes are not comparable until normalized to control (in yellow highlight on each sheet). Screen 1 and Screen 2 had averaged controls.

“DetailedDrugInfo.xlsx”

This file, modified from the small molecule file from Selleck Chem, shows detailed information for each of the small molecules in the library. The first column (A) shows the well of the plate that the small molecule was in. The second column (B) shows the small molecule’s unique identifier which can also be found in “AllScreenNotes.xlsx” in order to easily compare files. Column C shows the catalog number of the small molecule from Selleck Chem, and Column D shows the target of the small molecule. Columns E-I shows detailed information directly from Selleck Chem on the small molecule, including any known aliases of the compound and other targets.

SUPPLEMENTAL FIGURES

Supplemental Figure 1

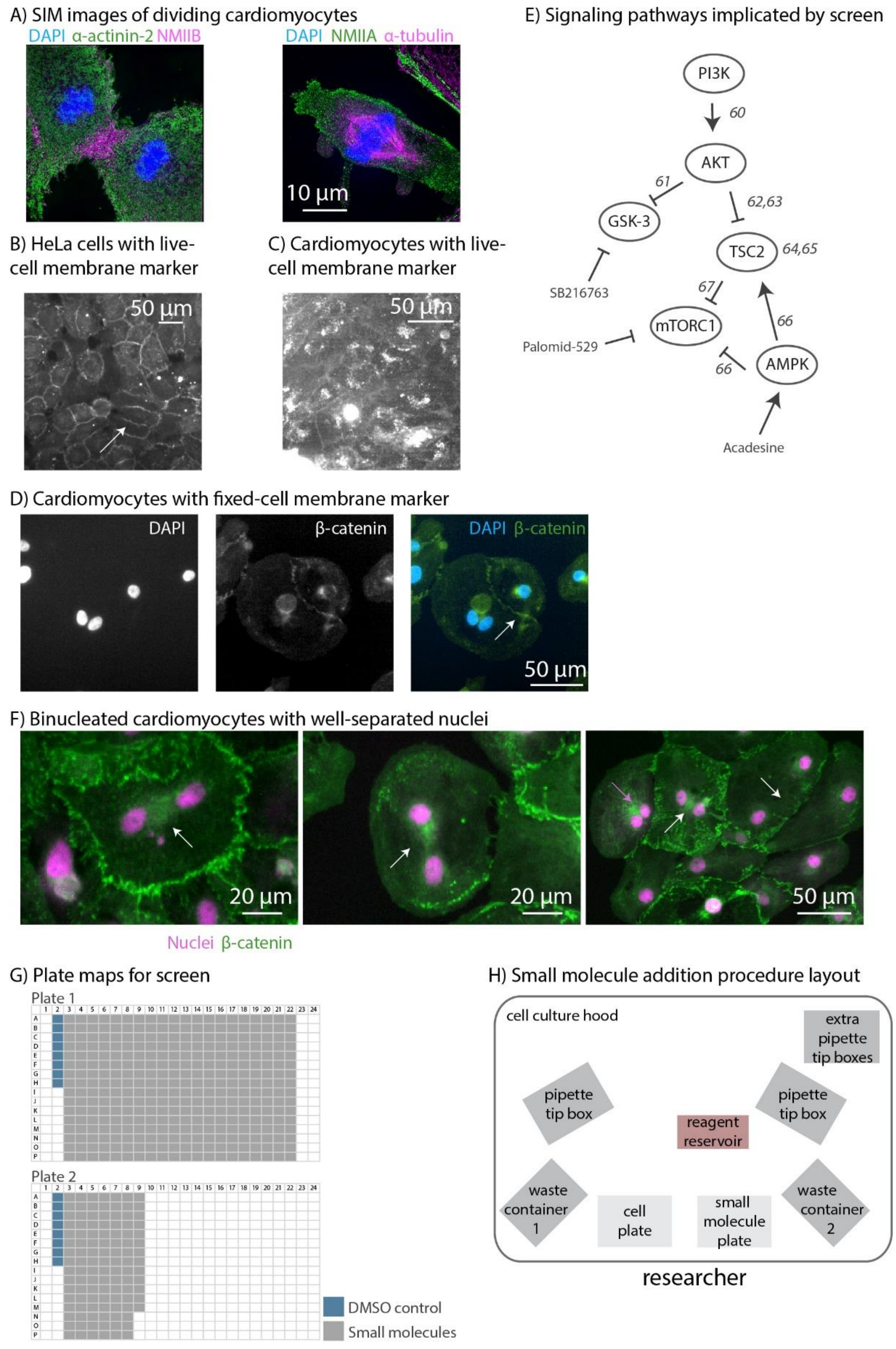

Supplemental Figure 2: Extended phenotypic examples of hiCMs treated with small molecules

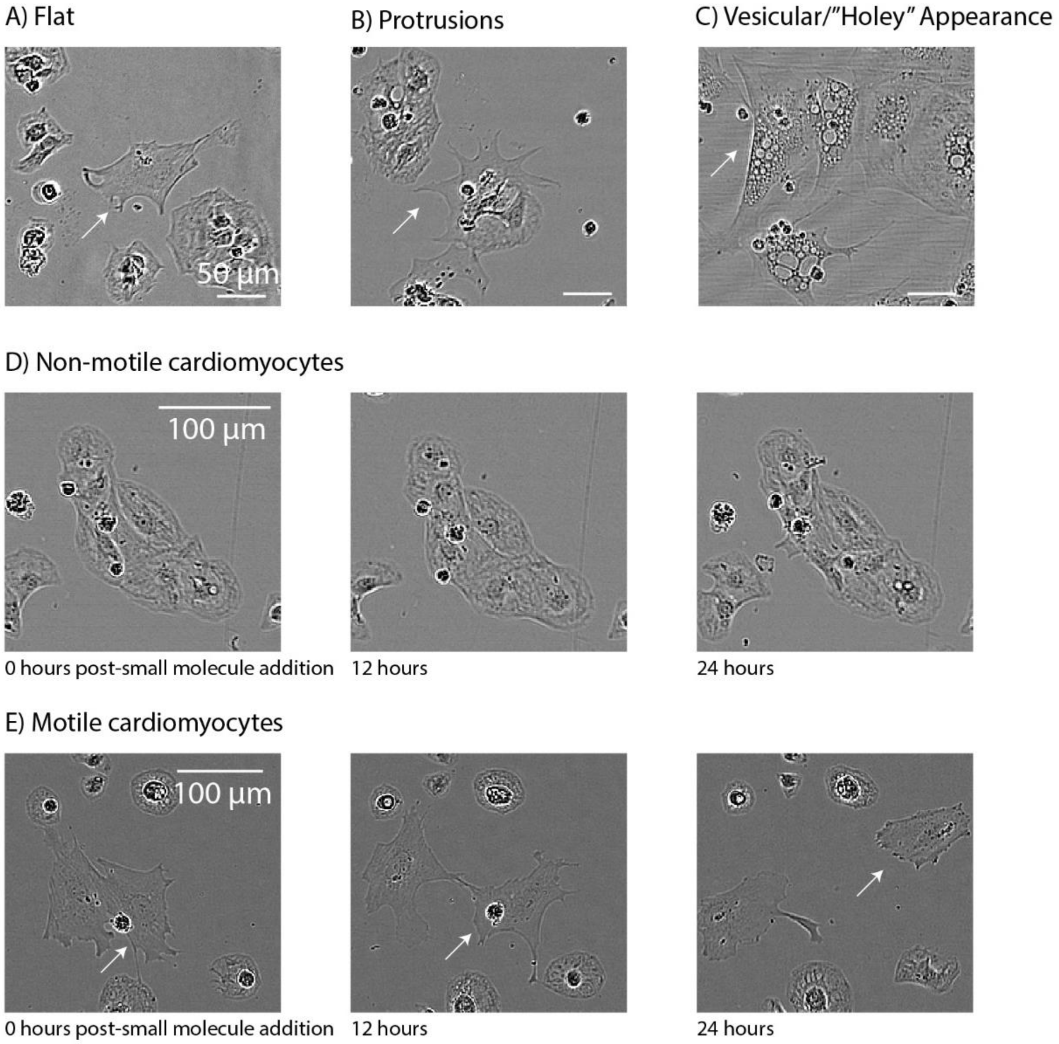

Supplemental Figure 3: Follow up of small molecules identified in screen

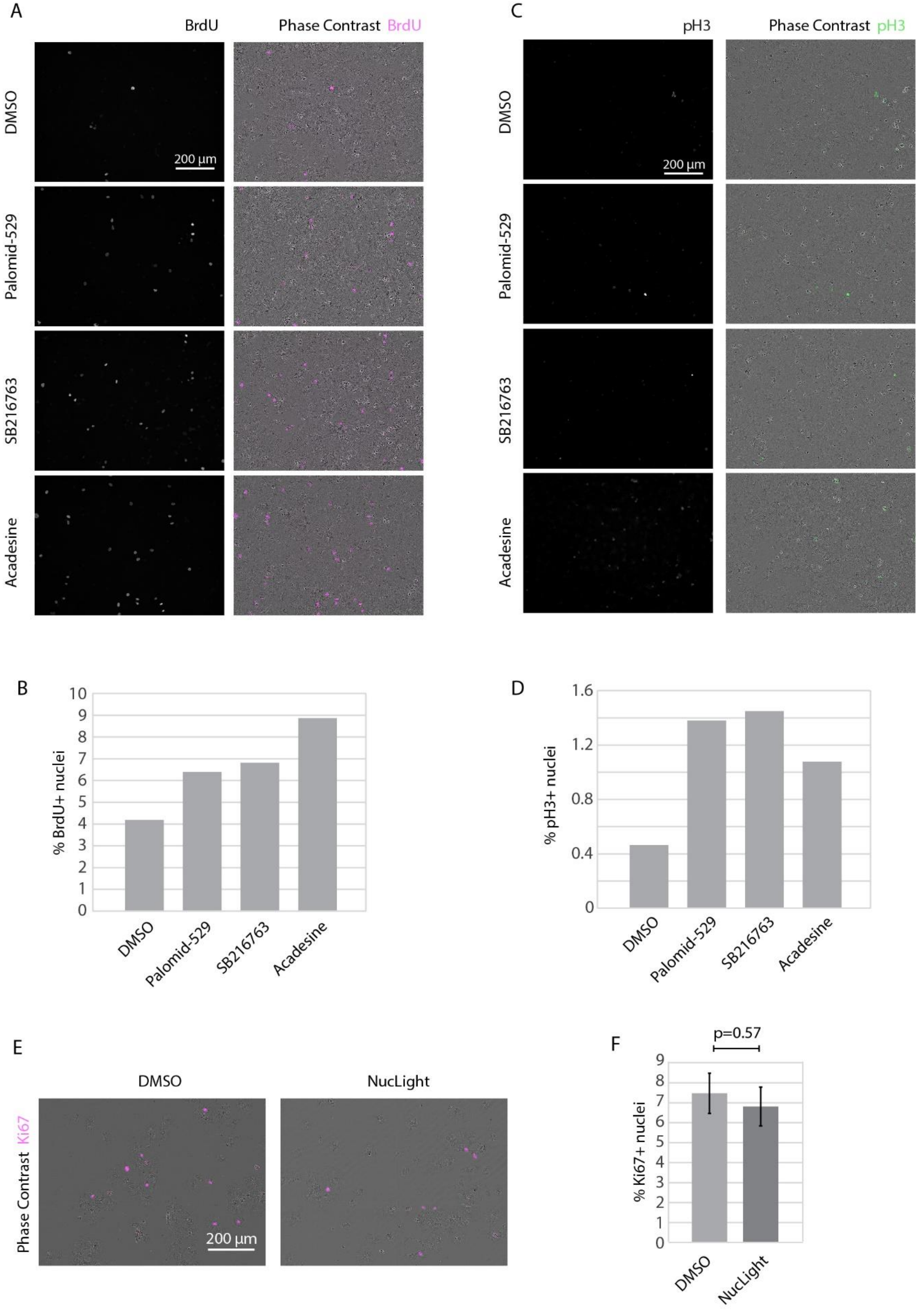

FIGURE LEGENDS

Figure S1

a) Structured illumination images of dividing cardiomyocytes 1 week post-plating.
b) HeLa cells with a live-cell membrane marker.
c) Cardiomyocytes with the same live-cell membrane marker as S1B. Poor localization may be attributed to the nature of rapid turnover of the plasma membrane in muscle cells[67]
d) Representative widefield image of a binucleated hiCM and a mononucleated hiCM, clearly separated by β-catenin-positive cell-cell adhesions.
e) Signaling pathways implicated in screen results.
f) Representative examples of binucleated cells with well-separated nuclei. White arrows: well-separated nuclei. Magenta arrow: binucleated cell with close nuclei.
g) Plate maps used in this manuscript.
h) Layout of cell culture hood during small molecule addition

Figure S2

A) Example of a cardiomyocyte which appears more flat (arrow) than a normal cardiomyocyte (below the flat cardiomyocyte). Small molecule: BYL719
B) Example of a cardiomyocyte with protrusions. Small molecule: AZD1480
C) Example of a cluster of cardiomyocytes that appear to have several vesicles throughout the cell, giving the cells a “holey” appearance. Small molecule: LY2603618
D) Montage of a stationary cardiomyocyte. Small molecule: Apatinib
E) Montage of motile cardiomyocytes. Small molecule: AS-252424

Figure S3

A) Phase contrast and BrdU localization in hiCMs given 5 µM of each small molecule identified in the screen and BrdU (Palomid-529, SB216763, and Acadesine) or DMSO control. Cells were treated with the small molecule for 48 hours before BrdU and small molecule treatment for 20 hours.
B) Quantification of percentage of BrdU-positive nuclei. N = 2443 cells
C) Phase contrast and pH3 localization in hiCMs given 5 µM of each small molecule identified in the screen and BrdU (Palomid-529, SB216763, and Acadesine) or DMSO control. Cells were in the presence of small molecules for three days.
D) Quantification of percentage of pH3-positive nuclei. N = 1882 cells
E) Phase contrast and Ki67 localization in control hiCMs and hiCMs treated with nuclight 1:4000 for 48 hours.
F) Quantification of percentage of Ki67-positive nuclei. N = 657 cells over 2 independent experiments.

SUPPLEMENTAL REFERENCES

1 Nakada, Y. *et al.* Hypoxia induces heart regeneration in adult mice. *Nature* **541**, 222-227, doi:10.1038/nature20173 (2017).

2 Tong, W., Xiong, F., Li, Y. & Zhang, L. Hypoxia inhibits cardiomyocyte proliferation in fetal rat hearts via upregulating TIMP-4. *Am J Physiol Regul Integr Comp Physiol* **304**, R613-620, doi:10.1152/ajpregu.00515.2012 (2013).

3 Vujic, A. *et al.* Exercise induces new cardiomyocyte generation in the adult mammalian heart. *Nat Commun* **9**, 1659, doi:10.1038/s41467-018-04083-1 (2018).

4 Han, C. *et al.* Acute inflammation stimulates a regenerative response in the neonatal mouse heart. *Cell Res* **25**, 1137-1151, doi:10.1038/cr.2015.110 (2015).

5 Eulalio, A. *et al.* Functional screening identifies miRNAs inducing cardiac regeneration. *Nature* **492**, 376-381, doi:10.1038/nature11739 (2012).

6 Tian, Y. *et al.* A microRNA-Hippo pathway that promotes cardiomyocyte proliferation and cardiac regeneration in mice. *Sci Transl Med* **7**, 279ra238, doi:10.1126/scitranslmed.3010841 (2015).

7 Diez-Cunado, M. *et al.* miRNAs that Induce Human Cardiomyocyte Proliferation Converge on the Hippo Pathway. *Cell Rep* **23**, 2168-2174, doi:10.1016/j.celrep.2018.04.049 (2018).

8 Chen, J. *et al.* mir-17-92 cluster is required for and sufficient to induce cardiomyocyte proliferation in postnatal and adult hearts. *Circ Res* **112**, 1557-1566, doi:10.1161/CIRCRESAHA.112.300658 (2013).

9 Huang, W. *et al.* Loss of microRNA-128 promotes cardiomyocyte proliferation and heart regeneration. *Nat Commun* **9**, 700, doi:10.1038/s41467-018-03019-z (2018).

10 Yang, Y. *et al.* MicroRNA-34a Plays a Key Role in Cardiac Repair and Regeneration Following Myocardial Infarction. *Circ Res* **117**, 450-459, doi:10.1161/CIRCRESAHA.117.305962 (2015).

11 Porrello, E. R. *et al.* Regulation of neonatal and adult mammalian heart regeneration by the miR-15 family. *Proc Natl Acad Sci U S A* **110**, 187-192, doi:10.1073/pnas.1208863110 (2013).

12 Hu, Y. *et al.* Suppression of miRNA let-7i-5p promotes cardiomyocyte proliferation and repairs heart function post injury by targetting CCND2 and E2F2. *Clin Sci (Lond)* **133**, 425-441, doi:10.1042/CS20181002 (2019).

13 Burton, P. B., Raff, M. C., Kerr, P., Yacoub, M. H. & Barton, P. J. An intrinsic timer that controls cell-cycle withdrawal in cultured cardiac myocytes. *Dev Biol* **216**, 659-670, doi:10.1006/dbio.1999.9524 (1999).

14 Engel, F. B., Hsieh, P. C., Lee, R. T. & Keating, M. T. FGF1/p38 MAP kinase inhibitor therapy induces cardiomyocyte mitosis, reduces scarring, and rescues function after myocardial infarction. *Proc Natl Acad Sci U S A* **103**, 15546-15551, doi:10.1073/pnas.0607382103 (2006).

15 Pasumarthi, K. B., Kardami, E. & Cattini, P. A. High and low molecular weight fibroblast growth factor-2 increase proliferation of neonatal rat cardiac myocytes but have differential effects on binucleation and nuclear morphology. Evidence for both paracrine and intracrine actions of fibroblast growth factor-2. *Circ Res* **78**, 126-136 (1996).

16 Mohamed, T. M. A. *et al.* Regulation of Cell Cycle to Stimulate Adult Cardiomyocyte Proliferation and Cardiac Regeneration. *Cell* **173**, 104-116 e112, doi:10.1016/j.cell.2018.02.014 (2018).

17 Naqvi, N. *et al.* A proliferative burst during preadolescence establishes the final cardiomyocyte number. *Cell* **157**, 795-807, doi:10.1016/j.cell.2014.03.035 (2014).

18 Bersell, K., Arab, S., Haring, B. & Kuhn, B. Neuregulin1/ErbB4 signaling induces cardiomyocyte proliferation and repair of heart injury. *Cell* **138**, 257-270, doi:10.1016/j.cell.2009.04.060 (2009).
19 Reuter, S., Soonpaa, M. H., Firulli, A. B., Chang, A. N. & Field, L. J. Recombinant neuregulin 1 does not activate cardiomyocyte DNA synthesis in normal or infarcted adult mice. *PLoS One* **9**, e115871, doi:10.1371/journal.pone.0115871 (2014).
20 Polizzotti, B. D. *et al.* Neuregulin stimulation of cardiomyocyte regeneration in mice and human myocardium reveals a therapeutic window. *Sci Transl Med* **7**, 281ra245, doi:10.1126/scitranslmed.aaa5171 (2015).
21 D'Uva, G. *et al.* ERBB2 triggers mammalian heart regeneration by promoting cardiomyocyte dedifferentiation and proliferation. *Nat Cell Biol* **17**, 627-638, doi:10.1038/ncb3149 (2015).
22 Sharma, A. *et al.* Stage-specific Effects of Bioactive Lipids on Human iPSC Cardiac Differentiation and Cardiomyocyte Proliferation. *Sci Rep* **8**, 6618, doi:10.1038/s41598-018-24954-3 (2018).
23 Fang, R. *et al.* Sustained co-delivery of BIO and IGF-1 by a novel hybrid hydrogel system to stimulate endogenous cardiac repair in myocardial infarcted rat hearts. *Int J Nanomedicine* **10**, 4691-4703, doi:10.2147/IJN.S81451 (2015).
24 Chakraborty, S., Sengupta, A. & Yutzey, K. E. Tbx20 promotes cardiomyocyte proliferation and persistence of fetal characteristics in adult mouse hearts. *J Mol Cell Cardiol* **62**, 203-213, doi:10.1016/j.yjmcc.2013.05.018 (2013).
25 Malek Mohammadi, M. *et al.* The transcription factor GATA4 promotes myocardial regeneration in neonatal mice. *EMBO Mol Med* **9**, 265-279, doi:10.15252/emmm.201606602 (2017).
26 Jackson, T. *et al.* The c-myc proto-oncogene regulates cardiac development in transgenic mice. *Mol Cell Biol* **10**, 3709-3716 (1990).
27 Chaudhry, H. W. *et al.* Cyclin A2 mediates cardiomyocyte mitosis in the postmitotic myocardium. *J Biol Chem* **279**, 35858-35866, doi:10.1074/jbc.M404975200 (2004).
28 Zhang, Y., Mignone, J. & MacLellan, W. R. Cardiac Regeneration and Stem Cells. *Physiol Rev* **95**, 1189-1204, doi:10.1152/physrev.00021.2014 (2015).
29 Busk, P. K. *et al.* Cyclin D2 induces proliferation of cardiac myocytes and represses hypertrophy. *Exp Cell Res* **304**, 149-161, doi:10.1016/j.yexcr.2004.10.022 (2005).
30 Hassink, R. J. *et al.* Cardiomyocyte cell cycle activation improves cardiac function after myocardial infarction. *Cardiovasc Res* **78**, 18-25, doi:10.1093/cvr/cvm101 (2008).
31 Sun, Q., Zhang, F., Wafa, K., Baptist, T. & Pasumarthi, K. B. A splice variant of cyclin D2 regulates cardiomyocyte cell cycle through a novel protein aggregation pathway. *J Cell Sci* **122**, 1563-1573, doi:10.1242/jcs.047738 (2009).
32 Williams, S. D., Zhu, H., Zhang, L. & Bernstein, H. S. Adenoviral delivery of human CDC5 promotes G2/M progression and cell division in neonatal ventricular cardiomyocytes. *Gene Ther* **13**, 837-843, doi:10.1038/sj.gt.3302737 (2006).
33 Agah, R. *et al.* Adenoviral delivery of E2F-1 directs cell cycle reentry and p53-independent apoptosis in postmitotic adult myocardium in vivo. *J Clin Invest* **100**, 2722-2728, doi:10.1172/JCI119817 (1997).
34 Pasumarthi, K. B. & Field, L. J. Cardiomyocyte cell cycle regulation. *Circ Res* **90**, 1044-1054 (2002).
35 Kirshenbaum, L. A. & Schneider, M. D. Adenovirus E1A represses cardiac gene transcription and reactivates DNA synthesis in ventricular myocytes, via alternative pocket protein- and p300-binding domains. *J Biol Chem* **270**, 7791-7794 (1995).
36 Di Stefano, V., Giacca, M., Capogrossi, M. C., Crescenzi, M. & Martelli, F. Knockdown of cyclin-dependent kinase inhibitors induces cardiomyocyte re-entry in the cell cycle. *J Biol Chem* **286**, 8644-8654, doi:10.1074/jbc.M110.184549 (2011).

37 Chen, Z. *et al.* Ablation of periostin inhibits post-infarction myocardial regeneration in neonatal mice mediated by the phosphatidylinositol 3 kinase/glycogen synthase kinase 3beta/cyclin D1 signalling pathway. *Cardiovasc Res* **113**, 620-632, doi:10.1093/cvr/cvx001 (2017).
38 Kuhn, B. *et al.* Periostin induces proliferation of differentiated cardiomyocytes and promotes cardiac repair. *Nat Med* **13**, 962-969, doi:10.1038/nm1619 (2007).
39 Lorts, A., Schwanekamp, J. A., Elrod, J. W., Sargent, M. A. & Molkentin, J. D. Genetic manipulation of periostin expression in the heart does not affect myocyte content, cell cycle activity, or cardiac repair. *Circ Res* **104**, e1-7, doi:10.1161/CIRCRESAHA.108.188649 (2009).
40 Bassat, E. *et al.* The extracellular matrix protein agrin promotes heart regeneration in mice. *Nature* **547**, 179-184, doi:10.1038/nature22978 (2017).
41 Fan, Y. *et al.* Wnt/beta-catenin-mediated signaling re-activates proliferation of matured cardiomyocytes. *Stem Cell Res Ther* **9**, 338, doi:10.1186/s13287-018-1086-8 (2018).
42 Woulfe, K. C. *et al.* Glycogen synthase kinase-3beta regulates post-myocardial infarction remodeling and stress-induced cardiomyocyte proliferation in vivo. *Circ Res* **106**, 1635-1645, doi:10.1161/CIRCRESAHA.109.211482 (2010).
43 Zhou, J. *et al.* Loss of Adult Cardiac Myocyte GSK-3 Leads to Mitotic Catastrophe Resulting in Fatal Dilated Cardiomyopathy. *Circ Res* **118**, 1208-1222, doi:10.1161/CIRCRESAHA.116.308544 (2016).
44 Uosaki, H. *et al.* Identification of chemicals inducing cardiomyocyte proliferation in developmental stage-specific manner with pluripotent stem cells. *Circ Cardiovasc Genet* **6**, 624-633, doi:10.1161/CIRCGENETICS.113.000330 (2013).
45 Kim, Y. S. *et al.* Natural product derivative BIO promotes recovery after myocardial infarction via unique modulation of the cardiac microenvironment. *Sci Rep* **6**, 30726, doi:10.1038/srep30726 (2016).
46 Buikema, J. W., Zwetsloot, P. P., Doevendans, P. A., Sluijter, J. P. & Domian, I. J. Expanding mouse ventricular cardiomyocytes through GSK-3 inhibition. *Curr Protoc Cell Biol* **61**, 23 29 21-23 29 10, doi:10.1002/0471143030.cb2309s61 (2013).
47 Kerkela, R. *et al.* Deletion of GSK-3beta in mice leads to hypertrophic cardiomyopathy secondary to cardiomyoblast hyperproliferation. *J Clin Invest* **118**, 3609-3618, doi:10.1172/JCI36245 (2008).
48 Matsuyama, D. & Kawahara, K. Oxidative stress-induced formation of a positive-feedback loop for the sustained activation of p38 MAPK leading to the loss of cell division in cardiomyocytes soon after birth. *Basic Res Cardiol* **106**, 815-828, doi:10.1007/s00395-011-0178-8 (2011).
49 Engel, F. B. *et al.* p38 MAP kinase inhibition enables proliferation of adult mammalian cardiomyocytes. *Genes Dev* **19**, 1175-1187, doi:10.1101/gad.1306705 (2005).
50 Park, S. *et al.* Yes-associated protein mediates human embryonic stem cell-derived cardiomyocyte proliferation: Involvement of epidermal growth factor receptor signaling. *J Cell Physiol* **233**, 7016-7025, doi:10.1002/jcp.26625 (2018).
51 Xin, M. *et al.* Hippo pathway effector Yap promotes cardiac regeneration. *Proc Natl Acad Sci U S A* **110**, 13839-13844, doi:10.1073/pnas.1313192110 (2013).
52 Heallen, T. *et al.* Hippo pathway inhibits Wnt signaling to restrain cardiomyocyte proliferation and heart size. *Science* **332**, 458-461, doi:10.1126/science.1199010 (2011).
53 Xin, M. *et al.* Regulation of insulin-like growth factor signaling by Yap governs cardiomyocyte proliferation and embryonic heart size. *Sci Signal* **4**, ra70, doi:10.1126/scisignal.2002278 (2011).
54 Lin, Z. *et al.* Cardiac-specific YAP activation improves cardiac function and survival in an experimental murine MI model. *Circ Res* **115**, 354-363, doi:10.1161/CIRCRESAHA.115.303632 (2014).

55 Hara, H. *et al.* Discovery of a Small Molecule to Increase Cardiomyocytes and Protect the Heart After Ischemic Injury. *JACC Basic Transl Sci* **3**, 639-653, doi:10.1016/j.jacbts.2018.07.005 (2018).
56 von Gise, A. *et al.* YAP1, the nuclear target of Hippo signaling, stimulates heart growth through cardiomyocyte proliferation but not hypertrophy. *Proc Natl Acad Sci U S A* **109**, 2394-2399, doi:10.1073/pnas.1116136109 (2012).
57 Heallen, T. *et al.* Hippo signaling impedes adult heart regeneration. *Development* **140**, 4683-4690, doi:10.1242/dev.102798 (2013).
58 Lin, Z. *et al.* Pi3kcb links Hippo-YAP and PI3K-AKT signaling pathways to promote cardiomyocyte proliferation and survival. *Circ Res* **116**, 35-45, doi:10.1161/CIRCRESAHA.115.304457 (2015).
59 Liu, T. L. *et al.* Observing the cell in its native state: Imaging subcellular dynamics in multicellular organisms. *Science* **360**, doi:10.1126/science.aaq1392 (2018).
60 Gingras, A. C., Kennedy, S. G., O'Leary, M. A., Sonenberg, N. & Hay, N. 4E-BP1, a repressor of mRNA translation, is phosphorylated and inactivated by the Akt(PKB) signaling pathway. *Genes Dev* **12**, 502-513 (1998).
61 Cross, D. A., Alessi, D. R., Cohen, P., Andjelkovich, M. & Hemmings, B. A. Inhibition of glycogen synthase kinase-3 by insulin mediated by protein kinase B. *Nature* **378**, 785-789, doi:10.1038/378785a0 (1995).
62 Inoki, K., Li, Y., Zhu, T., Wu, J. & Guan, K. L. TSC2 is phosphorylated and inhibited by Akt and suppresses mTOR signalling. *Nat Cell Biol* **4**, 648-657, doi:10.1038/ncb839 (2002).
63 Manning, B. D., Tee, A. R., Logsdon, M. N., Blenis, J. & Cantley, L. C. Identification of the tuberous sclerosis complex-2 tumor suppressor gene product tuberin as a target of the phosphoinositide 3-kinase/akt pathway. *Mol Cell* **10**, 151-162 (2002).
64 Soonpaa, M. H., Kim, K. K., Pajak, L., Franklin, M. & Field, L. J. Cardiomyocyte DNA synthesis and binucleation during murine development. *Am J Physiol* **271**, H2183-2189, doi:10.1152/ajpheart.1996.271.5.H2183 (1996).
65 Pajak, L. *et al.* Sustained cardiomyocyte DNA synthesis in whole embryo cultures lacking the TSC2 gene product. *Am J Physiol* **273**, H1619-1627, doi:10.1152/ajpheart.1997.273.3.H1619 (1997).
66 Inoki, K., Zhu, T. & Guan, K. L. TSC2 mediates cellular energy response to control cell growth and survival. *Cell* **115**, 577-590 (2003).
67 Bhaskar, P. T. & Hay, N. The two TORCs and Akt. *Dev Cell* **12**, 487-502, doi:10.1016/j.devcel.2007.03.020 (2007).
68 Sharma, A. *et al.* Use of human induced pluripotent stem cell-derived cardiomyocytes to assess drug cardiotoxicity. *Nat Protoc* **13**, 3018-3041, doi:10.1038/s41596-018-0076-8 (2018).
69 Burnette, D. T. *et al.* A contractile and counterbalancing adhesion system controls the 3D shape of crawling cells. *J Cell Biol* **205**, 83-96, doi:10.1083/jcb.201311104 (2014).
70 Fenix, A. M. *et al.* Muscle-specific stress fibers give rise to sarcomeres in cardiomyocytes. *Elife* **7**, doi:10.7554/eLife.42144 (2018).
